# The effect depression levels in midwives have on burnout and their level of job satisfaction

**DOI:** 10.18332/ejm/137486

**Published:** 2021-07-07

**Authors:** Sümeyye Altiparmak, Ayşe N. Yilmaz

**Affiliations:** 1 Department of Midwifery, Faculty of Health Sciences, Inonu University, Malatya, Turkey; 2 Department of Midwifery, Faculty of Health Sciences, Fırat University, Elazığ, Turkey

**Keywords:** depression, emotional exhaustion, midwife, job satisfaction, exhaustion

## Abstract

**INTRODUCTION:**

This study aimed to determine the effect of depression levels in midwives on burnout and job satisfaction.

**METHODS:**

The sample of this descriptive study consisted of 322 midwives working in a province located in the eastern region of Turkey. Data were collected by using the Personal Information Form, Beck Depression Inventory (BDI), Maslach Burnout Inventory (MBI), and Minnesota Job Satisfaction Questionnaire (MJSQ) Short Form.

**RESULTS:**

The rate of midwives experiencing depression was found to be 9.6%. The mean score of MJSQ was 64.59±13.29, while the mean scores for the sub-dimensions of MBI were: emotional burnout 18.57±6.65, decrease in sense of personal accomplishment 21.65±3.93, and depersonalization 6.25±3.86. It was found that midwives who did not experience depression (90.4%) had a higher level of job satisfaction and a higher sense of personal accomplishment (p<0.05). On the other hand, midwives experiencing depression (9.6%) had higher levels of emotional exhaustion and depersonalization. It was found that there was a negative weak relationship between BDI and job satisfaction and personal accomplishment, and a positive weak relationship between BDI and emotional exhaustion (p<0.05).

**CONCLUSIONS:**

It was determined that midwives with depression have lower levels of job satisfaction and personal success, and experience emotional exhaustion more frequently. In addition, it was observed that as the level of depression decreased, job satisfaction and personal success increased significantly, whereas emotional exhaustion decreased.

## INTRODUCTION

The emotional state that occurs in the face of life events that are undesirable or disappointing in healthy individuals is called depression^1^. In addition, depression is much more severe than emotional reactions such as distress and grief, and negatively affects the life of the individual by disrupting their vital functions. It can also disturb sleep and appetite; tiredness and poor concentration are common^1,2^. Depression is common in society, in all age groups and various personal and vital factors including age, gender, socioeconomic level, education level, and marital status, may pose a risk for depression^2-7^.

Depression also affects the level of burnout in individuals. Burnout is defined as depersonalization, emotional exhaustion and low sense of personal accomplishment, observed in individuals who have an intense relationship with other people due to their profession^8^. Burnout is a condition that can be affected by sociodemographic and occupational variables^9^. Burnout syndrome is seen as a first-degree threat for individuals who work in intense conditions, and individuals with this syndrome may feel without energy to start a new day compared to other individuals^8,10^.

Especially in individuals working as healthcare professionals, intense workload, providing care for serious and terminally ill patients, experiencing problems in sharing duties in the workplace, shift hours, disruption of sleep, and economic problems cause work-related stress and tension^11-13^. Witnessing or hearing about a traumatic event may have a traumatic effect similar to experiencing that event directly. Frequent encounters with traumatic events may also cause burnout and various mental problems in healthcare professionals^12^. Burnout syndrome experienced in professional life leads to problems such as an increase in depressive complaints, impaired quality of life, fatigue, sleep disorders, irritability, decreased job satisfaction, and disruption of professionalism. It has been reported that depression is more common in healthcare professionals than in the general public^11-14^.

Midwives provide some of the most important healthcare services including prenatal care, delivery and postnatal care with strategies for the development, improvement and protection of maternal and child health. In addition, midwives play an important role in reducing maternal and infant mortality^15-17^. The fact that healthcare services focus on people, and require very careful and continuous work, makes the job satisfaction for those working in this field even more important. Midwives’ potential for providing qualified service in the field of healthcare depends on their job satisfaction as well as their professional scientific knowledge. Job satisfaction is also affected by individual, organizational, and environmental factors^10,15,16,18^. Midwives who experience dissatisfaction with their job may experience situations such as continuous complaint, feeling dismissal and indifference towards the job, and feel hopeless regarding the future of the profession. In addition, job dissatisfaction may result in slowdown or shutdown of services, decrease in work efficiency, significant absenteeism and delays, resignation, incompatibility between peers, and non-compliance with business rules and work orders^15,16,18^. This situation is also emphasized by the intrinsic and extrinsic sources of motivation that affect Herzbergs’ job satisfaction^19^.

It is thought that job satisfaction of midwives is necessary for both the happiness of midwives and the improvement of quality of the services provided by them, and therefore the health level of society. If job satisfaction levels of midwives are determined and the reasons for the decrease are defined, activities that will increase job satisfaction can be planned and implemented^15,18^. For this purpose, this study aims to evaluate the effect of depression levels in midwives on burnout and job satisfaction.

## METHODS

### Study design and sample

This study aimed to determine the effect of depression levels in midwives on burnout and job satisfaction, qualitatively. The study was conducted on midwives working in all hospitals and family health centers in a province in the east of Turkey between 15 and 30 October 2020. A total of 553 midwives work in the institutions where the study was conducted. A total of 322 midwives who voluntarily accepted to participate in the study were included in the study. Midwives working in family and community health centers work in the day shift between 08:00–16:00, and midwives working in the hospital work either a day shift 08:00–16:00 or night shift 16:00–08:00. All midwives work at least 40 hours per week.

#### Inclusion criteria

The midwives who have been working in midwifery for at least 1 year in order to have work experience, working actively at the time of the study, those who were without any diagnosed psychiatric health problem, and who have internet access and volunteered for the study were included in the study.

#### Exclusion criteria

The midwives who did not respond to all questionnaires were excluded from the study.

### Data collection tools

Personal Information Form with Beck Depression Inventory (BDI), Maslach Burnout Inventory (MBI) and Minnesota Job Satisfaction Inventory Short Form, which are frequently used in professional studies, were used to collect data.

### Personal information form

The personal information form was prepared by the researchers in line with the literature and consists of 14 questions, including 3 questions about the sociodemographic characteristics (age, educational status, income status) of the midwives and 11 questions about the job (years in the profession, duration, and type of work, etc.)^17,20^. This form was created by the researchers.

### Beck Depression Inventory (BDI)

Beck Depression Inventory was developed by Beck in 1961. BDI is used to determine the risk for depression and to measure the level of depressive symptoms and the changes in severity^21^. The validity and reliability studies were conducted in Turkish by Hisli in 1989^22^. It consists of 21 self-evaluation items scored between 0 and 3. Each item determines a behavioral pattern specific to depression in the last week. The total score that can be obtained from the scale varies between 0 and 63. The cut-off score was accepted as 17. Scores ≥17 are accepted as having depression symptoms. Cronbach’s alpha reliability coefficient of the scale was found to be 0.80^22^. In the present study, the Cronbach’s alpha reliability coefficient of the scale was 0.84.

### Maslach Burnout Inventory (MBI)

Maslach Burnout Inventory (MBI) was developed by Maslach and Jackson in 1981^23^. The inventory consists of 3 sub-dimensions including emotional burnout (EB), personal accomplishment (PA), and depersonalization (D). The scale includes a total of 22 items; 9 items evaluating emotional burnout, 8 items evaluating personal success, and 5 items evaluating depersonalization. The inventory was adapted to Turkish by Ergin^24^ in 1992 and some changes were made in the inventory. While the items in the original inventory were answered on a 7-point Likert scale, it was rearranged to 5 points in the Turkish version, since it was observed that a 7-point scale was not suitable for Turkish culture. The answers include: 0 Never, 1 Very rare, 2 Sometimes, 3 Most of the time, and 4 Always. The total score of MBI is obtained by the sum of the sub-dimension scores; EB and D sub-dimensions are scored as defined above, while PA sub-dimension is scored reversely (never: 4; always: 0). Therefore, low scores obtained from PA sub-dimension indicate burnout whereas high scores from the EB and D sub-dimensions indicate burnout. Cronbach’s alpha reliability coefficients were 0.83 for EB, 0.72 for Depersonalization, and 0.67 for decreased sense of PA^24^. In the present study, Cronbach’s alpha reliability coefficients of the scale were 0.87, 0.75, and 0.67, respectively.

### Minnesota job satisfaction questionnaire - short form

The short form of the Minnesota Job Satisfaction Questionnaire was developed by Davis^25^. The Turkish validity and reliability study of the scale was conducted by Ozdayi^26^ in 1991 and the items related to internal and external attitude factors in the long form were combined into a 20-item scale^26^. The scale has two sub-dimensions including internal job satisfaction and external job satisfaction.

Internal job satisfaction sub-dimension consists of 12 items that include the expressions ‘activity, freedom, change, moral values, helping others, authority, using their abilities, responsibility, creativity, appreciation, success, and respect in the society’.

External job satisfaction sub-dimension consists of 8 items that include the expressions ‘promotion opportunity, institutional policy and practices, colleagues, working conditions, salary, manager’s management approach, manager’s technical support, and job guarantee’.

The items are answered on a 5-point Likert type scale, and the answers given to each item are scored between 1 and 5, where 1 represents ‘Not satisfactory at all’ and 5 represents ‘Very satisfactory’. The higher scores indicate higher job satisfaction. The Cronbach alpha reliability coefficient for the internal consistency of the scale was 0.86^26^. In the present study, Cronbach’s alpha reliability coefficient of the scale was 0.92.

### Data collection

Before the data collection process, the responsible midwives, in the hospitals and family health centers where the study was conducted, were reached. The questionnaire forms prepared were sent online to the phones of the responsible midwives and the questionnaire forms were requested to be sent to the social media network of all midwives working in the clinic. The midwives who accepted to participate in the study were asked to fill in the questionnaire, which took approximately 5–10 minutes.

### Statistical analysis

The data were evaluated by using the SPSS 20.0 package program. In the statistical analysis Cronbach’s alpha, Pearson correlation analysis, and independent samples t-test, were used. The results were expressed as percentage distribution, mean and standard deviation. Results were evaluated at 95% confidence interval and a value of p<0.05 was considered to be statistically significant.

## RESULTS

The mean age of midwives was 37.82±9.07 years (range: 20–58), 50.3% of midwives were undergraduate/university graduates, and 57.5% were equal to their income and expenses, 50.6% worked in the provincial state hospital, 46.9% for 1–10 years, and 72.4% worked 40 hours a week. Midwives worked in an area related to obstetrics for an average of 9.14±6.23 years and 40.7% thought that the number of midwives in the service they work is not sufficient. In all, 89.1% of midwives stated that they liked their profession, 76.4% chose the unit they work willingly, 67.7% chose their profession willingly, 82.3% found their profession suitable for them, and 47.2% of the midwives stated that they were members of the trade union related to the profession (Supplementary file).

Depression status of midwives according to the Beck Depression Scale cut-off score is given in Table 1. While 90.4% of midwives were non-depressed, scores ≥17 points on the scale, 9.6% were depressed (Table 1).

**Table 1:** Depression status of midwives according to the cut-off score of the Beck Depression Inventory (N=322)

*Beck Depression Inventory*	*Cut-off score*	*n*	*%*
Depressed	<17	31	9.6
Non-depressed	≥17	291	90.4

Table 2 shows the range of possible scores in MJSQ and MBI sub-dimensions, the range of scores attained by the midwives, and the distribution of the mean scores. The range and mean scores attained by the midwives were: MJSQ 20–98 with mean 64.59±13.29; MBI emotional burnout sub-dimension 0–35 with mean 18.57±6.65; MBI decrease in the sense of personal accomplishment sub-dimension 10–32 with mean 21.65±3.93; MBI depersonalization sub-dimension 0–18 with mean 6.25±3.86.

**Table 2:** Range of possible scores in MJSQ and MBI sub-dimensions, the attained scores of the midwives, and the distribution of the mean scores (N=322)

*Scales*	*Range of possible scores*	*Range of attained scores*	*Mean±SD*
**MJSQ**	0–100	20–98	64.59±13.29
**MBI sub-dimensions**			
Emotional burnout	0–36	0–35	18.57±6.65
Personal accomplishment	0–32	10–32	21.65±3.93
Depersonalization	0–20	0–18	6.25±3.86

SD: standard deviation. MJSQ: Minnesota Job Satisfaction Questionnaire. MBI: Maslach Burnout Inventory.

The comparison of the mean scores of midwives in the sub-dimensions of MJSQ and MBI according to their depression experience is given in Table 3. It was found that midwives who did not experience depression experienced higher levels of job satisfaction. Midwives with depression attained higher scores in the MBI sub-dimensions of emotional exhaustion and depersonalization (t= -3.147, p=0.002 and t= -0.792 p=0.429, respectively). Midwives who did not experience depression attained higher scores in the MBI sub-dimension of decrease sense of personal accomplishment (t=3.084, p=0.002).

**Table 3:** Comparison of the mean scores of MJSQ and sub-dimensions of MBI according to the depression status of midwives (N=322)

*Scales*	*Depressed Mean±SD*	*Non-depressed Mean±SD*	t-test*and p**
**MJSQ**		65.62±12.83	t=4.402 **p=0.000**
**MBI sub-dimensions**			
Emotional burnout	22.09±6.68	18.19±6.54	t= -3.147 **p=0.002**
Personal accomplishment	19.61±4.19	21.87±3.84	t=3.084 **p=0.002**
Depersonalization	6.77±4.50	6.19±3.79	t= -0.792 p=0.429

* Independent samples t-test.

** p<0.001.

JSQ: Minnesota Job Satisfaction Questionnaire. MBI: Maslach Burnout Inventory.

The relationship between mean total scores of BDI, MJSQ, and sub-dimensions of MBI of midwives are given in Table 4. There was a statistically weak and negatively significant relationship between the BDI and the MJSQ mean scores, and as the level of depression increased, job satisfaction decreased significantly (r= -0.297; p=0.000). There was a statistically positive and weakly significant relationship between the BDI mean score and mean score attained from the emotional burnout sub-dimension, and as the level of depression increased, emotional burnout increased significantly (r=0.297; p=0.000). There was a statistically negative weakly significant correlation between the BDI mean score and the mean score attained from the sub-dimension of decreased personal accomplishment, and as the level of depression increased, the sense of personal accomplishment decreased significantly (r= -0.175; p=0.000). There was a statistically weak and insignificant relationship between the BDI mean score and the mean score attained from the depersonalization sub-dimension, and as the level of depression increased, depersonalization decreased significantly (r= -0.175; p=0.076) (Table 4).

**Table 4:** The relationship between of the mean total scores of BDI, MJSQ, and sub-dimensions of MBI

*Scales*	r*	*p*
BDI – MJSQ	-0.297	0.000**
BDI – emotional burnout	0.297	0.000**
BDI – personal accomplishment	-0.175	0.002***
BDI – depersonalization	0.099	0.076

* Pearson correlation analysis.

** p<0.001.

*** p<0.05.

BDI: Beck Depression Inventory. MJSQ: Minnesota Job Satisfaction Questionnaire. MBI: Maslach Burnout Inventory.

## DISCUSSION

It our study, 50.3% of the midwives were undergraduate, 50.6% were working in the provincial state hospital and 87.0% were working in the midwife position. In the study of Ucucu^27^, it was stated that 59.5% of midwives had an undergraduate and 72.2% were working in state and university hospitals. In the study of Baskaya^28^, it was found that 70.4% of midwives had an undergraduate degree. In our study, 72.4% of midwives were working 40 hours per week and 40.7% thought that the number of midwives in the service they work was not sufficient. In the study of Ucucu^27^, it was found that 49.8% of midwives work 40 hours a week. In the study of Baskaya^28^, it was reported that 69.3% of the midwives thought that the number of midwives was not sufficient.

In the literature, it has been stated that midwives who chose their profession willingly have higher levels of job satisfaction^29^. In our study, 89.1% of midwives liked their job, 67.7% had chosen their profession willingly, and 76.4% had chosen the unit they work willingly. In the study of Baskaya^28^, when midwives were asked why they had chosen the midwifery profession, 26.4% had chosen midwifery because they liked it, and 28.2% had chosen it because their families wanted it. Similar to our results, in the study of Ucucu^27^, 70.7% of the midwives preferred the unit they worked in. In the study of Toker et al.^9^, 60.3% of midwives stated that they liked their profession, 66.2% wanted to improve themselves, and 61.8% chose their profession willingly. In the same study, 47.1% of midwives stated that they chose the profession because of the easy job opportunity. In our study, 82.3% of midwives stated that they thought their profession was suitable for them. However, in the study of Ucucu^27^, this rate was 69.8%. This difference is thought to be related to personal variables.

In our study, the rate of midwives who scored higher than 17 points, which is the cut-off point of BDI, namely those who were not experiencing depression was 90.4%, while the rate of those experiencing depression was 9.6%. In the study of Yildirim and Hacihasanoglu^11^, on healthcare professionals, the rate of those experiencing depression was 16.4%. In a study conducted on midwives in the United Kingdom, it was found that one-third of the participants scored moderate or above in terms of depression, anxiety and stress^30^. In the study of Creedy^31^, on Australian midwives, it was reported that approximately 20% of midwives experienced significant depression, anxiety and stress symptoms.

In our study, midwives who did not experience depression experienced a higher level of job satisfaction. In the study of Creedy^31^, the midwives exhibited high levels of personal and work-related burnout. This situation shows that midwives were affected by burnout, depression, anxiety and stress symptoms frequently in their private and professional lives.

The mean score of midwives on MJSQ was 64.59±13.29. In our study, job satisfaction of midwives was at a medium level. It is thought that midwives cannot achieve a high level of job satisfaction due to factors including workload, insomnia, fatigue, intense stress, and the COVID-19 pandemic. In the study of Ucucu^27^ on midwives, the mean MJSQ score was 56.21±12.47. Tekin et al.^32^ in their study stated that the mean MJSQ score of midwives and nurses working in primary healthcare in Cankiri was 61.81±12.79. In the study of Erdogan^33^, the mean MJSQ score of nurses was 82.07±6.15 and the job satisfaction level was high. Our study is compatible with the literature. In addition, in our study, there was a weak negative significant relationship between the BDI and MBI mean scores, and as the level of depression increased, job satisfaction decreased significantly.

The effects of job satisfaction and motivational factors on the efficiency and effectiveness of midwives directly influences the quality of health care^33^. Midwifery is one of the occupational groups that closely deal with the physical, emotional and social problems of the people they serve. While dealing with these problems, midwives will be able to feel the needs of their own physical, social and psychological health. As a matter of fact, the tension resulting from the decrease in job satisfaction as a result of not meeting the needs can negatively affect the mental health of individuals and cause burnout^34^. Similar to this study, Taycan et al.^35^ found that depression and burnout rates were lower in those who were generally satisfied with their job. On the other hand, Creedy^31^ suggested that future studies should evaluate midwives to determine whether occupational burnout or personal factors affecting women cause or contribute to the development of depression.

In our study, we determined that there was a statistically significant weak positive correlation between the mean BDI and the emotional burnout sub-dimension of the MBI scores and as the depression level increased, emotional burnout increased significantly. In the study of Taycan et al.^35^, it was found that there was a positive significant correlation between the mean BDI and the emotional burnout sub-dimension scores of the nurses (r=0.343; p<0.01). In the study of Unver et al.^34^, it was determined that as the level of tension due to work increases, the level of emotional exhaustion and depersonalization increases significantly. In the study of Yildirim and Hacihasanoglu^11^ on healthcare workers, a weak positive correlation was found between the mean BDI and the emotional burnout sub-dimension scores (p<0.001).

In our study, there was a weak negative significant correlation between the mean BDI and the decrease in personal accomplishment sub-dimension of MBI scores, and as the depression level increased, the sense of personal accomplishment decreased significantly. Consistent with our study, Taycan et al.^35^ reported that a significant negative correlation was found between the mean BDI and decrease in personal accomplishment sub-dimension scores of nurses (r= -0.151; p<0.01). In the study of Yildirim and Hacihasanoglu^11^ on healthcare professionals, a statistically weak positive relationship was found between the mean BDI and decrease in personal accomplishment sub-dimension of MBI (p<0.001).

### Strengths and limitations

Our study has some strengths and limitations. There is no study examining depression, burnout and job satisfaction on midwives in the literature review. This is the strongest aspect of this work. Inclusion of midwives working in all family health centers and hospitals at the place where the study was conducted is another strength. But, only 60% of the midwives were reached and this is a limitation of the study. The criterion of ‘Doing the midwifery profession for at least 1 year’ among the recruitment criteria may have resulted in this. The work is also limited to midwives working in a province in eastern Turkey. For this reason, the results obtained from the study cannot be generalized to all midwives in Turkey.

## CONCLUSIONS

Midwives with depression have lower levels of job satisfaction and personal success and experience more emotional exhaustion. In addition, it was seen that as the level of depression increased, job satisfaction and personal success increased significantly, whereas emotional exhaustion decreased. Reducing the depression and burnout levels of midwives, who have important roles in terms of family and public health, and increasing their job satisfaction levels will increase both their happiness and the quality of the service provided, and the health level of the family and society will increase. Factors that cause depression and burnout should be determined in midwives, and interventions and support systems should be planned for these factors.

## CONFLICTS OF INTEREST

The authors have completed and submitted the ICMJE Form for Disclosure of Potential Conflicts of Interest and none was reported.

## FUNDING

There was no source of funding for this research.

## ETHICAL APPROVAL AND INFORMED CONSENT

Ethical approval for the study was obtained from Inonu University Medical Sciences Scientific Research and Publication Ethics Committee (Decision No: 2020/1160). An institutional approval was also obtained from the Provincial Health Directorate of the relevant province, before initiating the research. Participation was voluntarily, and participating midwives were informed for the aims of the research at the beginning of the questionnaire.

## DATA AVAILABILITY

Data sharing is not applicable to this article as no new data were created.

## PROVENANCE AND PEER REVIEW

Not commissioned; externally peer reviewed.
