# Supplementary material for: The effect depression levels in midwives have on burnout and their level of job satisfaction
Source: Eur J Midwifery. 2021 Jul 7;5:24. doi: 10.18332/ejm/137486 (PMC8265175; doi:10.18332/ejm/137486)
Supplement: Supplementary file 1 [file EJM-5-24-s1.pdf]

Table. Distribution of Descriptive Characteristics of Midwives (n = 322)

**Descriptive Characteristics**	**Mean±SD**	
Age (years)	37.82 ± 9.07 (range: 20-58)	
Working time in the field related to obstetrics (years)	9.14 ± 6.23	
	**n**	**%**
**Educational level**		
High school	20	6.2
Associate Degree	118	36.6
Undergraduate/ University	162	50.3
Master's degree	22	6.8
**Income Status**		
Less than my revenues	104	32.3
The revenue is equal to the expense	185	57.5
More than my revenue	33	10.2
**Working Unit**		
County state hospital	65	20.2
Provincial State Hospital	163	50.6
University Hospital	26	8.1
Family and Community Health Center	68	21.1
**Total Term of Duty**		
From 1 to 10 years	151	46.9
Between 10-20 years	69	21.4
More than 30 years	102	31.7
**Weekly Working Hours**		
40 hours	233	72.4
41 hours and above	89	27.6
**Adequate Number of Midwives in the Service Worked**		
Yes	95	29.5
Partially	96	29.8

No	131	40.7
**Loving the Profession**		
Yes	287	89.1
No	35	10.9
**Selecting the Unit of Study Willingly**		
Yes	246	76.4
No	76	23.6
**Willingly Choosing The Profession**		
Yes	218	67.7
No	104	32.3
**Finding the Profession Suitable for You**		
Yes	265	82.3
No	57	17.7
**Membership in an association or union related to the profession**		
Only Association	18	5.6
Only Union	152	47.2
Association and Union	10	3.1
None	142	44.1
**Total**	**322**	**100.0**

SD: Standard Deviation

© 2021 Altiparmak S. and Yilmaz A. N.
